# Economic burden of moderate to severe burns and its association with health-related quality of life of Nigerian women

**DOI:** 10.1186/s12905-021-01232-5

**Published:** 2021-02-28

**Authors:** Anthonia U. Chinweuba, Ifunanya S. Chinweuba, Faith C. Diorgu, Nneka E. Ubochi, Chinwe S. Ezeruigbo, Kenneth B. Wasini, Anthonia I. Nnabuenyi

**Affiliations:** 1 Department of Nursing Sciences, Faculty of Health Sciences and Technology, College of Medicine, University of Nigeria, Enugu Campus, Enugu, Nigeria; 2 Department of Medicine and Surgery, College of Medicine, Chukwuemeka Odumegwu Ojukwu University, Awka, Anambra State Nigeria; 3 Department of Nursing Sciences, University of Port Harcourt, Rivers State, Port Harcourt, Nigeria; 4 Department of Nursing Science, Ebonyi State University, Abakaliki, Ebonyi State Nigeria; 5 Faculty of Nursing Sciences, College of Health Sciences, Niger Delta University, Wilberforce Island, Bayelsa State Nigeria; 6 School of Nursing, University of Nigeria Teaching Hospital, Enugu, Enugu State Nigeria

**Keywords:** Burns, Economic burden, Economic status, Health expenditure, Hospitalization, Quality of life

## Abstract

**Background:**

Burns cases are frequent in Nigeria hospitals, however, literature on its economic burden and the association with health-related quality of life (HRQOL) of women in Nigeria is scarce. This study determined the burden of hospitalization after burns on women’s economic status and its associated HRQOL.

**Methods:**

This was a three-month cross-sectional study of female patients ≥ 25 years, treated of mixed or full thickness burns in four teaching hospitals in south-east Nigeria, discharged between September–November, 2018. Study instruments were participants’ case notes for socio-demographic and disease history, interviewer-administered questionnaires, namely-economic-burden-of-burns questionnaire and English version of the EuroQol Five-Dimensions-Three-Level Health Questionnaire for Nigeria. Data were collected on second- or third-day post-discharge through home visits or phone calls. This lasted for 13 weeks.

**Results:**

A total of seventy-three female patients with burn were successfully enrolled. Most participants were married, fairly educated, mainly traders or housewives. Thirty-four (46.6%) had subjective estimated family monthly income below NGN 50,000 (low economic status). Participants’ average monthly income reduced drastically after hospitalization. Their average family monthly income was NGN110,439 (USD307), while their average total expenses incurred during hospitalization was NGN691,093 (USD1,920). Almost all (93.2%) had at least one surgical intervention during management. Their average length of hospital stay was 35.4 days; eleven consequently lost their job. Many had moderate to severe economic burden of treatment; only eleven could bear all the treatment expenses independently. Anxiety/depression and pain/discomfort were common problems reported, However, these Euroqol dimensions varied according to their SES, education and occupation. Women in the low economic class were more inclined to poor HRQOL (Mean ± SD VAS = 53.33 ± 17.619) than women in high economic class (Mean ± SD VAS = 76.67 ± 21.794).

**Conclusion:**

Burns places high level of economic burden on women and unfortunately, Nigerian government’s commitment to healthcare of burns patients is low. The long course of hospitalization and economic depletion impact negatively on the women’s HRQOL. Based on these findings, we recommend that government parastatals create special trust fund for burns treatment and the National Health Insurance Scheme be restructured for more accessibility.

## Background

Burn injury is a major public health concern. It is characterized as skin and/or other organic tissue injury caused primarily by extreme heat. Burn injury is ranked fourth in common traumatic conditions after traffic accident, falls and interpersonal violence [1]. An estimated 180,000 global annual deaths occur due to fire-related burn injuries, 95% of which were in low- and middle-income countries [2]. Non-fatal burns have been noted to be leading cause of morbidity, prolonged hospitalization and disability [2], which may affect one’s health-related quality of life (HRQOL). Health-related quality of life represents a self-report of one’s perceived feeling of satisfaction with life, comfort and ability to realize one’s life potentials. HrQoL is closely linked with responsibilities of an individual.

Cases of burn injury are frequently recorded in Nigeria hospital. Although few literatures have evaluated the impact and cost of managing burns in Nigeria [3, 4], there is still dearth of literature on economic burden of burns and its association with HRQOL of burns, especially female patients in this part of the world where women are exposed to a lot of socio-economic challenges [5, 6]. Enyioha [7] noted fast changing economic roles of African women as the world turns into a global village. These responsibilities translate to the amount of burden or difficulty the woman experiences. Severity of burn injury, depression, post-traumatic stress symptoms, inadequate social support, and loss of employment after hospitalization have been identified as predictors of poor HRQOL after burns [8], Economic hardship in women is directly proportional to their health trajectories [9]. Older women with lower socio-economic status and higher family/career responsibilities have lower HRQOL [10]. Similarly, homemakers who are often financially dependent experience more economic hardship than the career women that are relatively less financially dependent [11].

The economic drain on the patient’s purse due to burns treatment is huge especially in developing countries, with a mean hospital cost of about USD2,766 (NGN1,002,675) and a range from USD143 to USD33,566 (NGN51,480–NGN12,083,760) [12–14]. Studies in Nigeria have shown that the average length of hospital stay (LHS) for burn management was 19 days, the average daily cost of treatment per patient was USD47.74 [15] and the total cost of management was USD7,123.28 per patient [5]. However, Nigeria’s average daily income per person is less than USD1 [16] and health insurance scheme is almost non-existent [17–19]. The Nigerian National Health Insurance Scheme (NHIS) is a social security system launched formally in 2006 the aim of which is to improve the health of all Nigerians by increasing access to universal healthcare, providing healthcare at an affordable cost through various prepayment systems. This scheme is meant to provide equal access to health care across different income classes and reduce the out of pocket expenditure on healthcare by the insured through contributions by the insurer, usually the federal government [20]. This high cost of care has been attributed to series of surgeries and long hospitalization with accompanying huge out-of-pocket expenditures [4, 21–23].

The aim of the study was to determine the economic burden of hospitalization after burns on patients. Specific objectives were to: assess the cost of hospitalisation after burns; assess the sources of fund for medical and non-medical expenses available to the women; and, determine the women’s HRQOL based on the economic burden of burns management. It was hypothesized in this study that there is no significant association between the women’s socio-economic status and their HRQOL. This study will contribute to literature pools for economic evaluation of burns treatment.

## Methods

This was a three-month cross-sectional descriptive study of adult female patients admitted in hospitals, treated of burns and were discharged between 1st September and 30th November, 2018. Cross-sectional descriptive study had been used successfully in similar studies [10, 24]. The study was carried out in four teaching hospitals in southeast Nigeria. Inclusion criteria were: women ≥ 25 years, treated of mixed or full thickness accidental burns (≥ 30% total surface burn area according to the Rule of Nines for estimating burn percentage in adults), who were mentally stable and gave voluntary consent to participate.

**Fig. 1:**
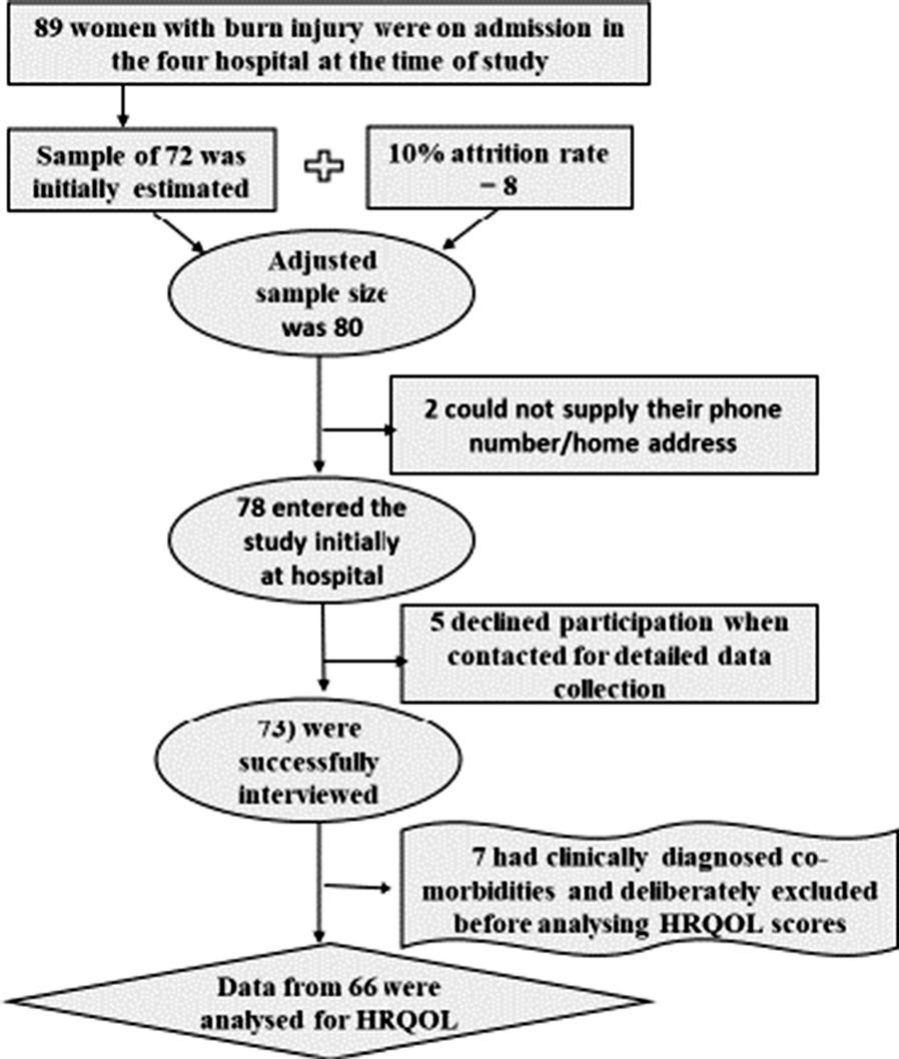
Flowchart of eligible patients and participants

A total of 89 women with burn injury were on admission in the four hospital at the time of study. A sample size of 72 women was initially estimated using the Creative Research Systems survey software’s sample size calculator formula: ss = (Z^2^*(p)*(1-p))/C^2^; where: Z = 1.96, p = proportion of target population (women ≥ 25 years old, estimated to have > 30% total surface burn area) (expressed as 0.5), C = Confidence Interval (0.04 ± 4) [25]. Applying the adjusted sample size formula for anticipated 10% attrition rate: *x* = ss/1-f (where *x* is adjusted sample size; ss is original sample size; and, f is estimated non-response rate) [26], this initial sample size estimate was adjusted from 72 to 80 representing about 89.9% of the population. The flow chart of eligible patients and participants is presented in Fig. 1.

### Instrument for data collection

In this study, HRQOL is based on scores obtained on the European quality of life—five dimensions three levels instrument (EuroQol-5D-3L). Participants’ case notes and interview-administered questionnaire developed in three parts were used. Part A collected eleven data on socio-demographic profile of participants and their clinical parameters namely: phone number, residential address, age, marital status, parity, education, occupation, estimated family monthly income (FMI), pre-hospital treatment, number of surgeries done and other co-morbidities. Part B was researcher-developed economic burden of burns questionnaire (EBB-Q) adapted from Deshpande, Puri, Vora, Shende and Choudhary [27] with nine items. Deshpande and colleagues formulated the EBB-Q to guide their data collection in a retrospective study on how families of patients cope with socio-economic burden of burns. The socio-demographic profile of participants, their clinical parameters and the researcher-developed EBB-Q are provided as Additional File 1. To determine the existence of economic gradient in HRQOL of women who had burns, participants were assigned social status based on their estimated FMI. Thus, women with estimated FMI less than NGN50,000 (< USD139) were classified as low economic status (LES), NGN50,000–NGN300,000 (USD139 to USD834)/month–middle economic status (MES), and more than NGN 300,000 (> USD834)/month–high economic status (HES). Cost of hospitalization include direct medical and other (subjective) non-medical expenditures incurred as a result of the burn treatment. Direct medical costs include costs of medications and consumables, surgeries, investigations, blood products, nursing care and bed space. Indirect costs include dietary cost, transportation costs by caregivers and other incidental expenses. Direct costs are billable; hence, direct costs were obtained from respective patient’s case notes. Indirect costs were estimates provided by the respondent during interview. Part C was EuroQol Five-Dimensions-Three-Level (EQ-5D-3L) Health Questionnaire for face-to-face and telephone methods. English version of the EQ-5D-3L for Nigeria and license to use same were obtained from the Euroqol Foundation through formal email. The EQ-5D-3L is a generic, preference-based measure of health with five dimensions, namely: mobility, self-care, usual activities, pain/discomfort, and anxiety/depression; and three levels of health state coded 1–3. Where: 1 = no problem, 2 = Moderate problem and 3 = extreme problem. It also contains a EuroQol visual analogue scale (EQ VAS) that requires the respondent to indicate the point on the scale where she would put her ‘own health state today’ ranging from ‘0’ (the worst health she can imagine) to ‘100’ (the best health she can imagine). Validity of the EQ-5D-3L in burns patients was established by Oster, Willebrand, Dyster-Aas, Kildal and Ekselius [28]. The Head of Department of Health Administration and Management, University of Nigeria, Nsukka was requested to evaluate the readability, relevance and feasibility of the EBB-Q and the socio-demographic profiles /clinical parameters. Also, a professor in Medical-Surgical Nursing in the Department of Nursing Sciences, University of Nigeria, Nsukka and a plastic surgeon from Burns and Plastic Unit of the National Orthopaedic Hospital, Enugu were presented with the instrument and requested to help in ascertaining that all relevant items were included in the EBB-Q and socio-demographics. All necessary corrections were subsequently effected. For internal consistency reliability test of the instruments, copies were administered to eight newly discharged burns patients at the Burns and Plastic female ward of the University of Nigeria Teaching Hospital, Ituku-Ozalla between 4 and 11th of August, 2018, representing 10% of the sample size, after due consent. Applying split-half method, the scores were computed using Cronbach Alpha reliability test. Reliability coefficient (r) of 0.88 for Part A, 0.79 for Part B, and 0.93 for Part C were considered satisfactory.

As some of the participants might not communicate effectively in English Language, an Ibo linguist was requested to translate the instrument to the local dialect (*Ibo*); another linguist was asked to translate the *Ibo* version to English Language without recourse to the original instrument. The translated English Language version was correlated with the original and discrepancies resolved in the local dialect version before use on the participants.

### Ethical consideration

Ethical approval to conduct the study was obtained from the Health Research Ethics Committee of the National Orthopaedic Hospital, Enugu (IRB/HEC Number: RET/313/111/988). In addition, administrative permit was sought from Heads of Nursing Services and Ward Heads of female burns and plastic wards of the respective hospitals. Written informed consent was obtained from participants before enrolment. Purpose of the study and role of participants were fully explained to the prospective participants prior to the consent. They were also assured of anonymity and confidentiality in data collection and use. Only women who gave consent were enrolled into the study.

### Procedure for data collection

With the assistance of clinicians in the female burns and plastic wards of the hospitals, researcher approached prospective participants already on admission. Purpose of the study and role of participants were explained to them while they were assured of confidentiality of information and non-maleficence. They were informed that researcher would visit them at their home two to three days after discharge from hospital for data collection. Cell phone numbers were exchanged with consenting patients to enhance communication. Data were collected at two points in time as follows:

Point 1, participant’s socio-demographic and disease history were elicited from their case notes at the time of discharge. Participants were identified using serial numbers assigned to their hospital file numbers and cell phone numbers (where available) for confidentiality. Thereafter, participants were asked to indicate if they would prefer to be visited physically at home or to have telephone call interview for data collection after discharge home. Two could not supply their phone number and/or home address and were excluded.

Point 2: Interviewer contacted participants between the 2nd and 3rd day after returning home for face-to-face or telephone call interview as pre-arranged. Only six opted for telephone calls. Member of the team visited the remaining seventy-two at their homes. For face-to-face method, interviewer established rapport, arranged for comfortable seats for the two, placed a copy of the questionnaire in front of him/her and gave a second copy to the participant for reference (where necessary). The interviewer read out contents of the questionnaire to the respondent item by item, using the preferred language version (English or *Ibo*) and entered participant’s answers directly into the questionnaire. They were asked questions about their ‘own health state today’ and requested to rate their health on a measuring scale, emphasizing honesty. To avoid bias, a question was only repeated word for word when participant asked for clarification. Interviewer’s personal explanations were avoided; instead, participant was asked to use her own interpretation and answer in a way that most closely resembled her thoughts about her ‘health state today’. The interview took 15–30 min to complete. Seventy-eight (78) patients entered the study initially while seventy-three (73) were successfully interviewed representing 93.6% of the population. The remaining five declined participation when contacted for detailed data collection. Data collection lasted for twelve weeks and five days.

### Statistical analysis

Data were analysed descriptively using frequencies, percentages, mean and standard deviation, and presented using contingency tables. Data collected on the estimated FMI and expenses due to burns and treatment were used to describe the economic burden of hospitalization after burns on the women’s economic life. Descriptive statistics of number, percentages, mean and standard deviation were used to report the health profile of the participants on each level of problem on each dimension of the EQ-5D-3L as affected by their condition [29]. At three-points scale, any mean ≥ 2 was considered a high level of problem and poor HRQOL. Level of health state of participants was established by determining the utility scores on the EQ-5D-3L scales according to their socio-demographic characteristics. To determine the economic burden of the burns, analysis of variance (ANOVA) was performed on the subjective degree of worsening of economic status due to burns and hospitalisation. ANOVA was used to analyse the association between the scores on the EQ-5D-3L VAS HRQOL of the women and their socio-economic status; and Tukey HSD applied for Post Hoc test of homogeneity of the scores where they existed. All statistical analyses were performed using the statistical package for social sciences version 23.0 computer software programme (SPSS inc., IL: Chicago, USA) at 95% confidence interval.

## Results

Seventy-three (73) post burns patients finally entered the study (Fig. 1).

### Patient characteristics

Table 1 showed that 34 (46.6%) out of the 73 women that entered the study had subjective estimated FMI below NGN50,000 (LES). Only 12 (16.4%) were classified as HES. Majority 45 (61.6%) were married. Half of the participants 37 (50.7%) were para 1–2, more than half of whom (20) had LES. As many as 46 (63.0%) were traders only four of whom were high-income earners. Majority had low educational attainment. Economic status appeared to be directly proportional to the women’s educational attainment as no participant with higher education had income below NGN50,000;

**Table 1:** Socio-demographic characteristics of participants *n* = 73

Personal characteristics	N	%
*Age (years)*		
25–34	28	38.4
35–44	26	35.6
≥ 45	19	26.0
*Marital status*		
Married	45	61.6
Single	18	24.7
Widow	10	13.7
*Parity*		
Nulliparous	18	24.7
Para 1–2	37	50.7
Para 3–4	8	11.0
≥ 5	10	13.7
*Occupation*		
Civil Servant	4	5.5
Self employed	13	17.8
Trader	46	63.0
homemaker	10	13.7
*Education*		
No formal	18	24.7
Primary	27	37.0
Secondary	19	26.0
Higher	9	12.3
*Socio-economic status*		
Low (< NGN 50,000)	34	46.6
Middle (NGN50,000-NGN300,000)	23	31.5
High (> NGN 300,000)	12	16.4
Don’t Know	4	5.5

Eight (11.0%) participants had received treatment elsewhere before reporting to the current hospital. Seven had one or more diagnosed co-morbidities. Significant post burn complications noted were unhealed wound/grafting 33 (45.2%) and contracture 21 (28.8%). More than half 39 (53.4%) had two or more surgical interventions during management; only 5 (6.8%) did not undergo surgery (Table 2).

**Table 2:** Other clinical history of participants *n* = *73*

Personal characteristics		Total	%
Any treatment for the burns prior to present hospitalization	Yes	8	11.0
	No	65	89.0
Other co-morbidities (single or in combination)	Hypertension	6	8.2
	Diabetes mellitus	4	5.5
	Asthma	1	1.4
	Actual number of participants with co-morbidities	7	9.6
Post burns status	Infection	6	8.2
	Contracture	21	28.8
	Itching at site of burn	14	19.2
	Unhealed wound/grafting	33	45.2
	Hypertrophic scar	5	6.8
Number of surgeries done	None	5	6.8
	1	29	39.7
	≥ 2	39	53.4

### Monthly income and cost of hospitalization

The women’s average subjective monthly income prior to admission reduced drastically after hospitalization. With the support of participants’ spouse and/or other family member(s) their average FMI before the burns marginally dropped at the end of hospitalization. The subjective average expenditure per day was NGN11,542 (USD32). With 35.4 days average LHS, the average total expenses incurred during hospitalization based on hospital bill at discharge and other estimated non-medical expenses related to hospitalization was NGN691,093 (USD1,920) (Table 3).

**Table 3:** Estimated monthly income and expenses due to hospitalization

		Self	Spouse/others in family	Total
Average income per month	Before injury	NGN48,388 (USD134)	NGN62,051 (USD172)	NGN110,439 (USD307)
	During admission	NGN23,750 (USD66)	NGN59,612 (USD166)	NGN83,362 (USD234)
Average expenses (N)^†^	Subjective average expenditure per day	–	–	NGN11,542 (USD32)
	Average medical expenses (A)	–	–	NGN549,142 (USD1,526)
	Average of other (non-medical) expenses (B)	–	–	NGN134,287 (USD373)
	Average total expenses (A + B)	–	–	NGN691,093 (USD1,920)
Average LHS		–	–	35.4 days

^**†**^Dollar equivalents are approximated to whole numbers (at NGN360/USD exchange rate)

**Fig. 2:**
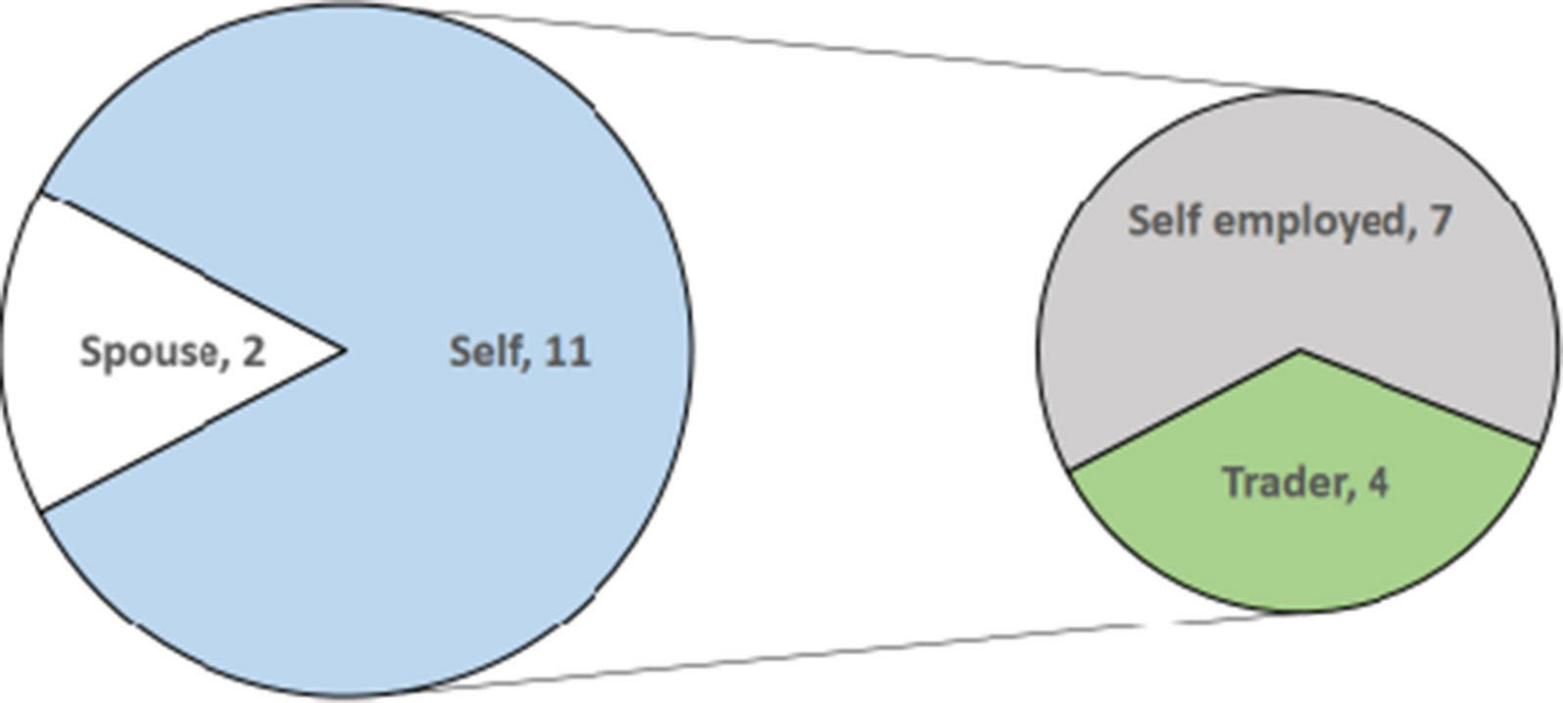
Loss of job as a result of hospitalization

Eleven women and two caregivers respectively lost their job as a result of hospitalization. Out of these, seven were self-employed while four were traders (Fig. 2).

### Source of fund

**Fig. 3:**
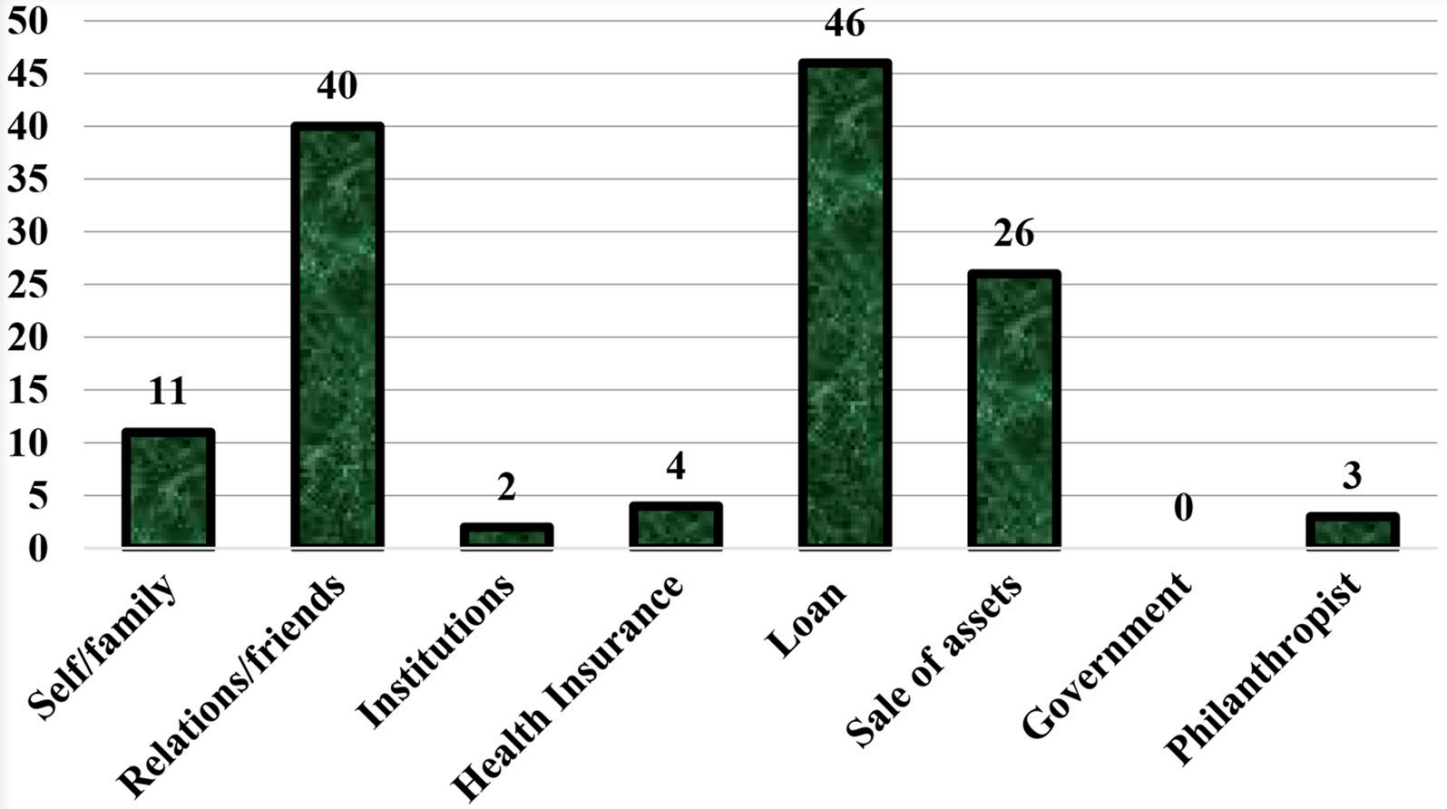
Source of fund for medical and non-medical expenses

As indicated in Fig. 3, only 11 participants could bear all expenses involved in their care at family levels, whereas more than half were assisted by relations and friends. As many as 46 (63.0%) sought loans from other sources for their treatment expenses. Twenty-six (35.6%) sold; while only 4 (5.5%) accessed the National Health Insurance Scheme (NHIS) for part payment of their cost of hospitalization. Not all values were exclusive as some participants had more than multiple source of fund.

### Economic burden

Half of the participants 37 (50.7%) had their economy moderately worsened following hospitalisation, while 23 (31.5%) had severe degree of worsening. However, analysis of variance of the scores showed no significant difference in the degree of economic worsening the women had post hospitalization based on their economic status (F = 1.101; p = 0.355) (Table 4).

**Table 4:** ANOVA of subjective degree of economic worsening due to hospitalisation *n* = *73*

Degree of economic worsening	N	Economic status				ANOVA result		
		Low 34 (%)	Middle 24 (%)	High 10%)	Don't know 5 (%)	Sum of squares	df, F	Sig
Severe	23 *(31.5)*	15 *(65.2)*	5 *(21.7)*	1 *(4.3)*	2 *(8.7)*	2.801	3 1.101	0.355
Moderate	37 *(50.7)*	15 *(40.5)*	11 *(29.70*	8 *(21.6)*	3 *(8.1)*			
A little	9 *(12.3)*	2 *(22.2)*	6 *(66.7)*	1 *(11.1)*	–			
Not at all	4 *(5.5)*	2 *(50.0)*	2 *(50.0)*	–	–			

### Participants’ health-related quality of life

**Fig. 4:**
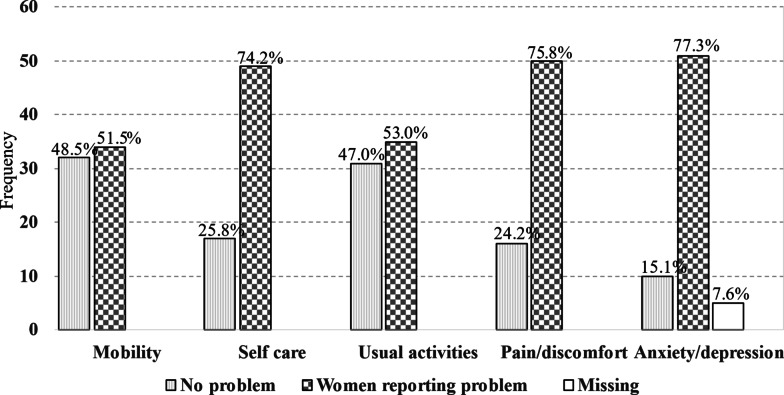
EQ-5D-3L frequencies reported by participants by dimension and level

Data from the seven (7) participants with clinically diagnosed co-morbidities (three participants had combine co-morbidities) were deliberately excluded before analysis to eliminate the possible confounding effect of these co-morbidities on their HRQOL scores; hence, data from 66 participants were analysed. Analysis of the participants’ report on the EQ-5D-3L by dimension and level (Fig. 4) showed that the least of their problems were mobility and performance of usual activities. About three quarters of the respondents each reported anxiety/depression 51 (77.3%), pain/discomfort 50 (75.8%) and problems with self care 49 (74.2%).

Table 5 showed that anxiety/depression was the greatest problem reported by the women. However, the anxiety/depression varied significantly according to their marital status, SES, occupation and education (p < 0.05). They reported problems with mobility and self-care irrespective of their marital status, parity, SES, occupation and education. Specifically, younger women had less problem with mobility while the older women had higher problems (p = 0.000). Similarly, the women’s report on pain/discomfort differed significantly according to their SES (p = 0.000), education (p = 0.000) and occupation (p = 0.002). With overall EQ-5D mean score of 2.04, that is > 2, participants’ HRQOL was considered as generally poor.

**Table 5:** EQ-5D-3L utility scores dimensions of participants’ HRQOL according to their socio-demographic characteristics*n* = *66*

EQ-5D	Levels	Socio-demographic distribution				P
		Age n (%)				
		25–34 (23)	35–44 (24)	> 45 (19)	Missing	
Mobility	No problems	21 (91.3)	8 (33.3)	3 (15.8)	–	.000*
	Patients reporting problems	2 (8.7)	16 (66.7)	16 (84.2)	–	
Self-care	No problem	21 (91.3)	8 (33.3)	3 (15.8)	–	.006*
	Patients reporting problems	2 (8.7)	16 (66.7)	16 (84.2)		
Usual activities	No problems	15 (65.2)	10 (41.7)	6 (31.6)	–	.077
	Patients reporting problems	8 (34.8)	14 (58.3)	13 (68.4)	–	
Pain/Discomfort	No pain or discomfort	8 (34.8)	5 (20.8)	3 (15.8)	–	.330
	Reported pain/discomfort	15 (65.2)	19 (79.2)	16 (84.2)	–	
Anxiety/depression	Not anxious/depressed	5 (21.7)	3 (12.5)	2 (10.5)	–	.457
	Anxious/depressed	17 (73.9)	19 (79.2)	15 (78.9)	–	
	Missing	1 (4.3)	2 (8.3)	2 (10.5)	–	
	Mean(SD)	1.93 ± .802				

		Marital status				
		Married (40)	Single (17)	Widowed (9)		
Mobility	No problems	21 (52.5)	6 (35.3)	5 (55.6)	**–**	.457
	Patients reporting problems	19 (47.5)	11 (64.7)	4 (44.4)	**–**	
Self-care	No problem	11 (27.5)	2 (11.8)	4 (44.4)	–	.185
	Patients reporting problems	29 (72.5)	15 (88.2)	5 (55.6)	–	
Usual activities	No problems	21 (52.5)	5 (29.4)	5 (55.6)	–	.248
	Patients reporting problems	19 (47.5)	12 (70.6)	4 (44.4)	–	
Pain/Discomfort	No pain or discomfort	10 (25.0)	4 (23.5)	2 (22.2)	–	.982
	Reported pain/discomfort	30 (75.0)	13 (76.5)	7 (77.8)	–	
Anxiety/ depression	Not anxious/depressed	7 (17.5)	3 (17.6)	0	–	.018*
	Anxious/depressed	32 (80.0)	13 (76.5)	6 (66.7)	–	
	Missing	1 (2.5)	1 (5.9)	3 (33.3)	–	
	Mean(SD)	1.53 ± .728				

		Parity				
		Nulliparous (16)	1–2 (32)	3–4 (7)	> 5 (11)	
Mobility	No problems	10 (62.5)	13 (40.6)	3 (42.9)	6 (54.5)	.529
	Patients reporting problems	6 (37.5)	19 (59.4)	4 (57.1)	5 (45.5)	
Self-care	No problem	5 (31.2)	7 (21.9)	1 (14.3)	4 (36.4)	.667
	Patients reporting problems	11 (68.8)	25 (78.1)	6 (85.7)	7 (63.6)	
Usual activities	No problems	9 (56.2)	13 (40.6)	2 (28.6)	7 (63.6)	.368
	Patients reporting problems	7 (45.8)	19 (59.4)	5 (71.4)	4 (36.4)	
Pain/Discomfort	No pain or discomfort	7 (45.8)	6 (18.8)	0	3 (27.3)	.107
	Reported pain/discomfort	9 (56.2)	26 (81.2)	7 (100.0)	8 (72.7)	
Anxiety/depression	Not anxious/depressed	5 (31.2)	3 (9.4)	0	2 (18.2)	.119
	Anxious/depressed	11 (68.8)	27 (84.4)	7 (100.0)	6 (54.5)	
	Missing	0	2 (6.2)	0	3 (27.3)	
	Mean(SD)	2.20 ± .996				

		Socio-economic status				
		Low (33)	Middle (20)	High (9)	Don’t know (4)	
Mobility	No problems	16 (48.5)	10 (50.0)	5 (55.6)	1 (25.0)	.793
	Patients reporting problems	17 (51.5)	10 (50.0)	4 (44.4)	3 (75.0)	
Self-care	No problem	6 (18.2)	7 (35.0)	3 (33.3)	1 (25.0)	.557
	Patients reporting problems	27 (81.8)	13 (65.0)	6 (66.7)	3 (75.0)	
Usual activities	No problems	11 (33.3)	13 (65.0)	6 (66.7)	1 (25.0)	.064
	Patients reporting problems	22 (66.7)	7 (35.0)	3 (33.3)	3 (75.0)	
Pain/Discomfort	No pain or discomfort	2 (6.1)	8 (40.0)	6 (66.7)	0	.000*
	Reported pain/discomfort	31 (93.9)	12 (60.0)	3 (33.3)	4 (100.0)	
Anxiety/ depression	Not anxious/depressed	1 (3.0)	5 (25.0)	4 (44.4)	0	.000*
	Anxious/depressed	29 (87.9)	15 (75.0)	5 (55.6)	2 (50.0)	
	Missing	3 (9.1)	0	0	2 (50.0)	
	Mean(SD)	1.76 ± .913				

		Occupation				
		Civil Servant (4)	Self employed (12)	Trader (41)	Housewife (9)	
Mobility	No problems	1 (25.0)	7(58.3)	21 (51.2)	3 (33.3)	.529
	Patients reporting problems	3 (75.0)	5 (41.7)	20 (48.8)	6 (66.7)	
Self-care	No problem	1 (25.0)	1 (8.3)	13 (31.7)	2 (22.2)	.452
	Patients reporting problems	3 (75.0)	11 (91.7)	28 (68.3)	7 (77.8)	
Usual activities	No problems	4 (100.0)	5 (41.7)	18 (43.9)	4 (44.4)	.191
	Patients reporting problems	0	7 (58.3)	23 (56.1)	5 (55.6)	
Pain/Discomfort	No pain or discomfort	3 (75.0)	6 (50.0)	7 (17.1)	0	.002*
	Reported pain/discomfort	1 (25.0)	6 (60.0)	34 (82.9)	9 (100.0)	
Anxiety/ depression	Not anxious/depressed	2 (50.0)	4 (33.3)	4 (9.8)	0	.023*
	Anxious/depressed	2 (50.0)	8 (66.7)	33 (80.5)	8 (88.9)	
	Missing	0	0	4 (9.7)	1 (11.1)	
	Mean(SD)	2.63 ± .7.355				

		Education				
		No formal (15)	Primary (27)	Secondary (13)	Higher (11)	
Mobility	No problems	8 (53.3)	11 (40.7)	6 (46.2)	7 (63.6)	.625
	Patients reporting problems	7 (46.7)	16 (59.3)	7 (53.8)	4 (36.4)	
Self-care	No problem	4 (26.7)	4 (14.8)	5 (38.5)	4 (36.4)	.341
	Patients reporting problems	11 (73.3)	23 (85.2)	8 (61.5)	7 (63.6)	
Usual activities	No problems	5 (33.3)	10 (37.0)	7 (53.9)	9 (81.8)	.049*
	Patients reporting problems	10 (66.7)	17 (63.0)	6 (46.2)	2 (18.2)	
Pain/discomfort	No pain or discomfort	0	3 (11.1)	3 (23.1)	10 (90.9)	.000*
	Reported pain/discomfort	15 (100.0)	24 (88.9)	10 (76.9)	1 (9.1)	
Anxiety/depression	Not anxious/depressed	0	1 (3.7)	1 (7.7)	8 (72.7)	.000*
	Anxious/depressed	12 (80.0)	25 (92.6)	11 (84.6)	3 (27.3)	
	Missing	3 (20.0)	1 (3.7)	1 (7.7)	0	
	Mean(SD)	2.20 ± 1.007				
Grand Mean		2.04				

*Significance

Analysis of the reports on the EQ-5D-3L VAS ‘health state today’ (Table 6) showed a mean(± SD) of 57.12 ± 19.436. Specifically, as many as 15 participants reported an EQ VAS of 50—which incidentally was the median of the scores. The women’s EQ VAS ‘health state today’ varied significantly according to their SES (p < 0.002). The scores appeared to improve as the SES increased; thus, women of LES had mean (± SD) VAS score as low as 53.33 ± 17.619, median = 50; which increased to 76.67 ± 21.794, median = 90 for the HES.

**Table 6:** Participants’ reports on the EQ VAS according to their SES *n* = *66*

EQ VAS score (n)		Economic Status				P^†^
	Low 33 (%)	Middle 20 (%)	High 9 (%)	Don’t know 4 (%)	Total	
10	–	–	–	–	0	.002*
20	–	–	–	–	0	
30	5 (15.2%)	3 (15.0%)	1 (11.1%)	2 (50.0%)	11	
40	7 (21.2%)	–	0	1 (25.0%)	8	
50	8 (24.3%)	6 (30.0%)	1 (11.1%)	–	15	
60	5 (15.2%)	4 (20.0%)	0	1 (25.0%)	10	
70	3 (9.1%)	3 (15.0%)	0	–	6	
80	3 (9.1%)	4 (20.0%)	2 (22.2%)	–	9	
90	2 (6.1%)	0	5 (55.6%)	–	7	
100	–	–	–	–	0	
Mean (± SD) VAS	53.33 ± 17.619	58.00 ± 16.416	76.67 ± 21.794	40.00 ± 14.142	57.12 ± 19.436	
Median	50	60	90	35	50	

^†^1-Way ANOVA P value

*Significant

Multiple comparison of the scores on the EQ-5D-3L HRQOL using Tukey HSD Repeated Mean test show that the variations in SES were less for anxiety but were unequal guaranteeing Type 1 error (Table 7).

**Table 7:** Tukey HSD post-hoc test of homogeneity of the scores on EQ-5D-3L and EQ VAS of HRQOL

Quality of life scale	SES	Subset for alpha on the EQ-5D-3L = 0.05		
		1	2	3
Pain/discomfort	High SES (9)	1.556	–	–
	Middle SES (20)	1.700	–	–
	Low SES (33)	2.303	–	–
	Don't know (4)	2.250	–	–
Anxiety/depression	High SES (9)	1.667	–	–
	Middle SES (20)	1.950	1.950	–
	Low SES (33)	–	2.636	2.636
	Don't Know (4)	–	–	3.250
EQ VAS Scale	High SES (9)	3.222	–	–
	Middle SES (20)	2.600	–	–
	Low SES (33)	2.121	–	–
	Don't Know (4)	3.000	–	–

## Discussions

This study determined the burden of hospitalization after burns on women’s economic status and its associated HRQOL. The women had worsened economy post burn treatment due to high cost of hospitalization, loss of job and indebtedness. According to results of this study, a client spends as high as NGN691,093 (USD1,920) on the average on drugs, surgery, investigations, nursing care, bed space and other non-medical expenses (like feeding, transportation and procurement of toiletries). This is similar to $ 2,810 and $2,766 reported by Karimi [13] and Latifi et al. [14] respectively, but far below $7,123.28 estimated by Okafor et al. [4]. Meanwhile, the average LHS was 35.4 days—against the 19 days average found by Okafor et al. [4]; and FMI before the burns was just about 11.1% of the hospital expenses. Unfortunately, only 5.5% accessed the services of the NHIS. The near non-existed NHIS as already demonstrated [17–19] is thus supported by findings of this study. Poor health financing has pitiable implications for achievement of goals of universal health coverage in Nigeria.

Result of this study showed that although it was done in an urban setting, most of the participants were young, fairly educated, married women of low means, mainly traders or homemakers. Many of them had moderate to severe burden of treatment because of the high cost of treatment. Result indicated that as many as thirty-three were discharged home with open wounds and/or grafting possibly to avoid further accumulation of hospital bill. This finding directly reflects the low economic capacity of most of the women and the fact that most expenditure are out-of-pocket as they reported. Almost all the participants (68) had at least one surgical intervention during their burns management, meaning that burns patients will often need huge amount of money for operation and drugs deposits. In addition, they will require fund to procure the expensive modern dressing applications (like the Epigraft, Ex salt T7 and Therabond applications), high protein diet peculiar to burns treatment as well as pay for orthodox dressing change. If patient fails to pay for drugs, then drugs supply by the Pharmacy Department may be withdrawn. Unfortunately, antibiotics used in burns treatment are usually costly (like the injection Meronem that sells between N6,000.00 to N8,000.00 per vial) and procuring such may be challenging to the patient, resulting to more burden.

The estimated family income can only yield about 20.89% of the average total expenses incurred during hospitalization, all things being equal; meaning that the remaining 79.11% must be sourced elsewhere. Our study showed that there was worsening of economy for most of the women following hospitalization irrespective of their economic status, and that up to eleven women, mostly traders and self-employed women, lost their job due to hospitalization. Since many of the participants were women of low means, they were most likely to be sole traders operating easy and inexpensive business with unstable customers and finance. The reported drop in estimated subjective monthly income supports the fact that even the usual meagre income will stop flowing in when the woman is hospitalised. Consequently, many would secure loans from friends, money lenders, bank, cooperative society, and the likes (with interest in some situations) and/or mortgage/sell assets such as lands, automobile, power generating set, television, jewelleries and clothes to raise fund for offsetting the huge hospital bills. Borrowing, selling personal assets and sometimes, loss of job are catastrophic results of the injury. Resultant stress and financial hardship/burden for the women and their family will deepen the pre-existing low economic standard.

Further, the women’s HRQOL was poor as they reported anxiety/depression, self-care deficit and physical discomfort, thus aligning with Spronk [8]. Also, their EQ VAS and anxiety/depression scores differed significantly based on their occupation. Civil servants’ highest scores on the EQ VAS imply a better quality of life among them. Conversely, the highest anxiety/depression scores for the self-employed women mean poorer HRQOL. Civil servants work in domesticated organisations—they have little or no worry concerning the impact of hospitalization on their job because their job is secured. Self-employed women and traders, on the other hand, operate as ‘wild’ organisations because they always have to struggle for their own survival. Based on this, civil servants are more likely to have more positive assessment of their health state at any time than the self-employed and traders.

The women’s anxiety/depression dimension and EQ VAS varied based on their economic status. Although there was no significant difference in other EQ-5D-3L dimensions, women in the LES appeared to be more inclined to extreme anxiety or depression than the middle and high economic class. Tucker et al. [9] earlier reported this downward health trajectory for women with high economic hardship. With insufficient fund, making required deposits for treatments and surgeries, paying for expensive drugs, dressing packs and other medical and non-medical needs will become difficult.

It is understandable that a good number of the women reported ‘extreme pain or discomfort’ or ‘confinement to bed after long stay in hospital’ or inability to perform usual activities independently because burns is ordinarily characterised with pain. Some women may have requested for ‘pre-mature’ discharge (even with open wound and partially healed grafting) to reduce further accumulation of hospital bill. Patients are not supposed to be discharged until they are fully recovered and capable of performing most activities of daily living unassisted. Sick patients in hospital have their sick role; the health conditions are continuously monitored while their skilled care providers institute necessary actions. When the individual is discharged home before full recovery, self-dependence and perhaps care by unskilled caregivers becomes the available option. This comes with its risk of wound breakdown, infection, pain and other complications, further worsening the already poor HRQOL and high economic burden of treatment.

There was significant difference between the women’s age and their mobility, self-care and usual activities. As earlier observed by Chinweuba et al. [10], the older women had poorer mobility and less ability to perform usual activities than the younger women did. This is understandable because people are physically stronger, more active and better able to self-manage their problems at their youthful age and become weaker as they get old. Young women may also have less social responsibilities and fewer challenges with self-care. Increasing level of education tends to reduce the women’s anxiety levels probably because it (education) will enhance better understanding of one’s challenges, better-informed decision making and more focused health actions. Educated women will be better able to study and understand their problem and its management than their less educated counterparts.

Result showed significant difference in the women’s pain/discomfort dimension based on their parity. However, there was not any specific pattern in the differences. More studies may be required on this.

### Strengths and limitations

One strength of this study is the choice of patients with moderate to severe degrees of burn which allowed for full range assessment of impact of burn injury and its management. Women with varying economic status entered the study. This served the opportunity to measure how SES predict the HRQOL of the patients. Although this study was done in Nigeria, the evident data may be resourceful in countries with similar "burn" problems for health policy decision making. However, the study was constrained by paucity of related literature especially from other low- to medium-income countries for a better view and understanding of economic burden of hospitalization after burns. Non-medical expenses related to hospitalization were estimates that were liable to personal factors of the participants. The values, therefore, cannot be used reliably as standard in similar studies.

## Conclusion

Burns place high level of economic burden to the women who are predominantly of LES. It also has enormous negative impact on their quality of life. Many of them do not recover fully before leaving the hospital. The women’s HRQOL is directly proportional to their economic status and degree of economic burden. The long course of treatment and economic depletion through the huge out-of-pocket expenditure impact negatively on their HRQOL. Pain/discomfort, ‘reduced mobility and activities’ and anxiety/depression are commonly affected dimensions of the women’s HRQOL following burns. The women’s level of education is directly proportional to their HRQOL. Nigerian government’s commitment to healthcare of burns patients is low or non-existent.

Based on the findings, we recommended that State and national governments create special trust fund for treatment, or at least subsidize treatment of burns particularly for indigent patients with severe burns, where free treatment is not possible. The NHIS needs to be made more accessible to all categories of the citizenry. We also recommend formation of non-profit burns foundations by non-governmental agencies with special endowment funds to assist financing of burns treatment. Finally, female education should be made free and compulsory in Nigeria with secondary school as the minimum.

## Supplementary Information

**Additional file 1**. Economic Burden of Burns Questionnaire (EBB-Q).

## Data Availability

The datasets generated and/or analysed during the current study are not publicly available because some parts of the data contain confidential personal information of respondents and their family, but are available from the corresponding author on reasonable request.

## Abbreviations

ANOVA: Analysis of Variance
EBB-Q: Economic Burden of Burns Questionnaire
EQ VAS: Visual Analogue Scale
EQ-5D-3L: EuroQol Five-Dimensions-Three-Level
FMI: Family Monthly Income
HES: High Economic Status
HRQOL: Health-related Quality of Life
LES: Low Economic Status
LHS: Length of Hospital Stay
MES: Middle Economic Status
NGN: Nigeria Naira
NHIS: National Health Insurance Scheme
QALYs: Quality-Adjusted Life Years
USD: United States Dollar
WHO: World Health Organisation
