# Supplementary material for: Economic burden of moderate to severe burns and its association with health-related quality of life of Nigerian women
Source: BMC Womens Health. 2021 Feb 28;21:85. doi: 10.1186/s12905-021-01232-5 (PMC7916273; doi:10.1186/s12905-021-01232-5)
Supplement: Supplementary file 1 — Additional file 1. Economic Burden of Burns Questionnaire (EBB-Q). [file 12905_2021_1232_MOESM1_ESM.docx]

**Additional File 1**

**Part A: Respondent’s Socio-demographics**  Hospital file No

Please provide the following information about you:

1. Phone number …………………………….
2. Residential address ………………………….
3. Age last birthday …………………
4. Marital status
   1. Married
   2. Single
   3. Widow
5. Number of children
   1. None
   2. 1 – 2
   3. 3 – 4
   4. 5 and above
6. Education
   1. No formal
   2. Primary
   3. Secondary
   4. Higher
7. Occupation
   1. Civil Servant
   2. Self employed
   3. Trader
   4. Homemaker
   5. Any other, please specify ……………
8. Estimated family monthly income
   1. <NGN 50,000
   2. NGN50,000-NGN300,000
   3. NGN 300,000 and above
   4. Don’t Know
9. Have you had any treatment for the burns before coming to this hospital
   1. Yes
   2. No
10. How many surgeries did you have while on admission? ……………….
11. Please indicate if you have any of the following conditions before admission by ticking √ as applies to you:
    1. Hypertension
    2. Diabetes mellitus
    3. Asthma
    4. None

**Part B: Economic Burden of Burns Questionnaire (EBB-Q)**

Kindly read through the items numbered 1 to 7 and respond as they apply to your hospitalisation. Tick √ in the boxes as appropriate and write your response to each open-ended item.

1. Did you have any of these complications during and/or after your hospitalization for treatment of burn injury? Please tick only the applicable one(s) or go to question 2 if none applies to you.
   1. Infection
   2. Contracture
   3. Itching at site of burn
   4. Unhealed wound/grafting
   5. Hypertrophic scar
2. What is your estimated income per month ***before injury***? ……………….
3. What is the estimated income per month ***before injury*** of your spouse and/or other members of your family? ………………..
4. What is your estimated income per month ***during admission***? ………….
5. What is the estimated income per month ***during admission*** of your spouse and/or other members of your family? …………….
6. On the average, how much did you spend each day on other (non-medical) needs related to your treatment, such as dietary cost, transportation costs by caregivers, toiletries, call credits, other incidental expenses, etc.? ……………………..
7. How would you describe degree of economic worsening due to hospitalization?
   1. Severe
   2. Moderate
   3. A little
   4. Not at all

***Please do not bother providing responses to items 8 and 9***

**Billable expenses from patients’ records:**

1. Medical expenses (costs of medications and consumables, surgeries, investigations, blood products, nursing care and bed space) ………………………….
2. Length of hospital stay (in days) ………………
